# The impact of cathelicidin, the human antimicrobial peptide LL-37 in urinary tract infections

**DOI:** 10.1186/s12879-017-2901-z

**Published:** 2018-01-08

**Authors:** Ibrahim H. Babikir, Elsir A. Abugroun, Naser Eldin Bilal, Abdullah Ali Alghasham, Elmuataz Elmansi Abdalla, Ishag Adam

**Affiliations:** 1 College of Medical Laboratory Sciences, Microbiology Department, University of Khartoum, Khartoum, Sudan; 2 Faculty of Medical Laboratory Sciences, University of Science and Technology, Omdurman, Sudan; 3 Khartoum University Central Research Laboratory, University of Khartoum, PO Box 321, Khartoum, Sudan; 4 College of Medicine, Qassim University, Buraydah, Qassim Kingdom of Saudi Arabia

**Keywords:** Urinary tract infections, Cathelicidin, LL-37, Antimicrobial peptide, Vitek 2 System, Uropathogenic *E. coli*, Sudan

## Abstract

**Background:**

The defense mechanisms of the urinary tract are attributed mainly to the innate immune system and the urinary tract urothelium which represent the first line of defense against invading pathogens and maintaining sterility of the urinary tract. There are only a few publications regarding cathelicidin (LL-37) and a urinary tract infection (UTI). This study was done to investigate the plasma and urine levels of human LL-37 in patients with UTI.

**Methods:**

A case-control study was conducted at Omdurman Hospital, Sudan during the period from August 2014 to May 2017. The cases were patients with confirmed UTI and the controls were healthy volunteers without UTI. Sociodemographic and clinical data were obtained from each participant using questionnaires. Urine cultures and antimicrobial susceptibility were tested. Plasma and urine levels of LL-37 were determined using an enzyme-linked immunosorbent assay (ELISA) kit. SPSS (version 16.0) was used for analyses.

**Results:**

Cases and controls (87 in each arm) were matched according to their basic characteristics. Compared with controls, the median (inter-quartile) LL-37 level in plasma [2.100 (1.700–2.700) vs. 1.800 (1.000–2.200) ng/ml, *P* = 0.002] and in urine [0.900 (0.300–1.600) vs. 0.000 (0.000–1.000) ng/mg creatinine, *P* < 0.001] was significantly higher in cases. There was no significant difference in the median plasma [2.1 (1.7–2.9) vs. 2.000 (1.700–2.400) ng/ml, *P* = 0.561] and urine [0.850 (0.275–2.025) vs. 0.900 (0.250–1.350) ng/mg creatinine, *P* = 0.124]. The uropathogenic *Escherichia coli* (UPEC) was the predominant isolate, *n* = 38 (43.7%). LL-37 levels between the *E. coli* isolates and the other isolated organisms. There was no significant correlation between plasma and urine LL-37 levels (*r* = 0.221), even when the data of the cases were analyzed separately.

**Conclusion:**

LL-37 is notably increased among patients with UTI compared with normal control subjects. Severity of UTI increases the levels of LL-37. The increased level was not only in the patient’s urine, but has also been observed in the patient’s plasma. Detection of increased levels of LL-37 could help to differentiate subjects with suspected UTI. Accordingly, LL-37 could act as a good marker for diagnosing UTIs.

## Background

Urinary tract infection (UTI) is a major health problem as it is one of the most common bacterial infections. The predominant causal organism is *Escherichia coli* (*E. coli*)*.* The innate immune response to urinary pathogens can both control and predispose to subsequent recurrence of UTIs, at least for a significant proportion of patients [1, 2].

Increasing bacterial resistance to common antibiotics has been a growing public health concern [2, 3]. Emergence of multi-drug resistant microbes was seen as early as the 1950s as the result of antibiotic misuse or overuse [4, 5].

Although, there are many routes by which microbes can reach the urinary tract and the kidneys [6, 7], the defensive mechanisms of the innate immune system, in addition to the urinary tract urothelium, represent the first line of defense against these invading pathogens helping the urinary tract to remain sterile [1]. Dysfunction in these immune mechanisms may lead to acute disease, massive infection and tissue destruction, which may turn the ‘friendly’ host defense into troublesome and disturbing enemies and give rise to disease, in addition to long-term complications such as hypertension and chronic kidney disease [1, 8]. Progression to renal scarring and permanent impairment of renal function and tissue destruction may occur [9, 10].

In mammals, several defense mechanisms guard against the threat of infection, including the innate immune response and physical factors, such as urine flow, pH, and ionic composition together with expression of natural antimicrobial peptides (AMPs) [1, 8]. AMPs are considered the first line of innate immune defense against invading pathogens as they play a fundamental role in protection from prokaryotes [11]. At least two distinct groups of AMPs have been reported; the defensins and cathelicidin. In contrast to the multiple defensins (alpha, beta, and gamma), to date, only one cathelicidin has been found in humans [11–13]. The gene product is synthesized as a propeptide and is referred to as human cationic antimicrobial peptide-18 (hCAP-18/LL-37). hCAP-18 is the precursor molecule and its molecular weight is 18 Kd. In various tissues, hCAP-18 is cleaved by proteases to form two parts; the N-terminal (cathelin) and C-terminal part. The latter is further cleaved enzymatically to produce a 37 amino acid peptide which starts with two leucines, hence the name LL-37; LL-37 is considered the main biologically active broad spectrum antimicrobial agent [14, 15].

Due to the cationic nature of LL-37, an amphipathic α-helical peptide, it has broad spectrum antimicrobial activity against bacteria, fungi, viruses, and parasites, but notably, shows low toxicity to human cells. During inflammation, it has been identified as a potent chemoattractant for innate and adaptive immune cells [9, 16]. LL-37 has been shown to neutralize lipopolysaccharides (LPS), which are endotoxins of Gram-negative bacteria that are released upon cell death, by binding to them with high affinity [17, 18]. Additionally, LL-37 has been shown to inhibit the association of LPS with its receptor, suppressing LPS-induced apoptosis of endothelial cells [19], and to block the effects of flagellin and lipoteichoic acid on dendritic cells [20].

Attention and interest concerning endogenous defense has been stimulated by increasing concern regarding antimicrobial overuse. Enhancement of natural mechanisms will be an exciting novel therapeutic avenue in the management of UTIs [1, 21]. LL-37 has potent, direct antimicrobial activity at minimum inhibitory concentrations (MIC) than synthetically and traditionally used antimicrobial agents [22, 23]. Moreover, these peptides have potential activity against multiple drug-resistant microbes [23, 24]. Thus, AMPs or their derivatives potentially represent a new category of antimicrobial agents [23, 24].

Production of cathelicidin peptides by blood cells [4] and by the epithelial cells in the urothelium mucous membranes has been proposed as a natural innate immune response to maintain a normal sterile urinary tract and represents a candidate for a novel class of antimicrobials [25, 26]. Despite the mechanisms of the innate immune response, bacteria still cause UTIs. In contrast, involvement of the adaptive immune response to UTIs is poorly understood. However, it has been suggested that secretory immunoglobulin A (sIgA) antibodies inhibit bacterial colonization by lowering bacterial adherence to the mucosa antibodies during UTIs, even though serum production of antibodies is characteristically difficult to detect in UTIs [27].

To our knowledge, this is the first study on cathelicidin in Sudan. However, there are a few globally published data on LL-37 and UTI [2, 8, 21, 26]. The current study was conducted to investigate plasma and urinary levels of LL-37 in patients with (culture positive) UTI compared with healthy volunteers, and to add to the previous research on UTI in Sudan [28, 29].

## Methods

This is a case-control study was conducted at Omdurman Hospital, Sudan, during the period June–September 2014. Institutional review board approval was obtained from the Faculty of Medical Laboratory Sciences Research Ethics Review Board, University of Khartoum, Sudan. A signed written informed consent was obtained from all subjects or from the parents or guardians in case of the participants under the 16 years of age. Sociodemographic and clinical data were obtained from each participant using structured pre-tested questionnaires.

### Study setting and population

Consecutive patients with UTI symptoms who attended the referral clinic were approached to participate in the study. Cases were (males and females) those who met the inclusion criteria; signs and symptoms of UTI, willingness (the participant agreed to participate), and no other health problem or underlying disease. The controls were apparently healthy volunteers with no current or previous history of UTIs. Diabetic patients, pregnant women or subjects with anatomical or functional abnormalities of the urinary tract or subjects with underlying diseases were excluded from both the case and control groups.

For cases, an initial survey covered medical history, demographics and symptoms (dysuria, frequency, urgency, lower abdominal or suprapubic pain, fever, costovertebral tenderness [flank pain], nausea and vomiting), in addition to their culture positive results.

### Collection and processing specimens

Paired Blood and urine samples were collected from each participant. Under a septic technique, 5 ml of blood was withdrawn in Ethylene-diamine-tetra-acetic acid (EDTA) anticoagulant tube. The blood samples were directly tested for complete blood count (CBC) using Sysmex KX-21 N, and then carefully centrifuged (1500×g at 4 °C for 15 min) to separate plasma. Plasma specimens were stored at −80 °C. For urine specimens all participants were asked to provide a midstream urine sample according to the clean-catch procedure. Urine Samples were collected using a sterile screw capped wide mouth container and processed immediately. In the medical laboratory each urine sample was divided into two; the first half was stored at −80 °C and the second half was immediately inoculated on standard culture media. A standard quantitative (1 μL and 10 μL) loop was used to inoculate urine samples on to Cysteine Lactose Electrolyte Deficient (CLED) agar, MacConkey’s and Blood Agar (Oxoid, Basingstoke, UK). Plates were incubated aerobically at 35–37 °C for 24 h and the outcome was judged as significant/non-significant growth, or contaminated (discarded). The rest of urine was centrifuged (1500×g for 5 min) to prepare urine debris for direct microscopic examination for Red Blood Cells (RBCs), pus cells (leukocyturia), epithelial cell count, casts, crystals and parasitic infection if present. In the normal urine sediment a few count of RBCs, pus cells (0–5 per high power field, HPF) and epithelial cells may present. Epithelial cell count reported as “few,” “moderate,” or “many” per low power field (LPF).

A portion of the urine specimens was used for dipstick test rapid response urinalysis Reagent Strips (Combi-Screen PLUS, Roche, USA). For the control participants, nitrite and leukocyte esterase positivity were considered as a positive indicator for active infection and those were excluded from the study. When the patient had a nitrite and leukocyte esterase dipstick negative results, UTI confirmed by urine culture examination.

LL-37 analysis for both plasma and urine samples was performed using ELISA kit (HK321–02 HycultBiotech, Gmbh, Germany) as described elsewhere [30–32].

The bacterial identification and the antimicrobial susceptibility were done by using the fully automated VITEK-2 Compact System (see below).

### Measurements

Colony counting is the numerical cut-off estimation for the number of viable bacteria in a milliliter of uncentrifuged urine; it is a quantitative estimation that enables us to differentiate the true bacteriuria from urethral or vulval contamination, which may occur during collection of mid-stream or “clean-catch” urine [26, 28, 29]. Multiplication of microbes in the urinary system is defined by the presence of more than 10^5^ CFU/ml of urine, which is significantly diagnostic of UTI [26, 28, 30]. Significant UTI was defined as urine culture plates showing ≥10^5^ colony-forming units (CFU)/mL freshly voided urine. Referring to the cut-off of 10^5^ CFU/ml, a positive urine culture was identified as ≥10^5^ CFU/ml of one to two organisms from a clean-catch specimen [28, 29].

The bacterial identification and the antimicrobial susceptibility were done by using the fully automated VITEK-2 Compact System. Prior application of Vitek system clinically significant isolates were sub-cultured for purity and inoculated on specific plates (nutrient agar or blood agar), then incubated aerobically at 35–37 °C in 5% CO_2_. Isolated bacteria were differentiated according to their colonial morphology and gram stain. After overnight incubation, the bacterial colonies were used to prepare a standardized saline inoculum for the appropriate VITEK identification (ID) card. For identification of bacteria, special ID and sensitivity (AST) cards (BioMérieux) were used. Gram positive ID card: [GP Reference 21 342], gram Positive sensitivity card: [GP/AST-580 Reference 22 233], gram negative ID card: [GN Reference 21 341], and gram negative sensitivity card: [AST-N291 Reference 415 062]. All methods and techniques were conducted as described by the manufacturer. The VITEK-2 ID and AST cards were logged and loaded into the VITEK-2 Compact system. The VITEK-2 Compact system automatically reported and printed the results through software 06.01.

A total sample size of 87 participants in each arm of the study was calculated using a formula for the difference in the mean of the proposed variables (plasma and urine LL-37) that would provide 80% power to detect a 5% difference at α = 0.05 and which assumed that 10% of participants might not have complete data.

The urinary LL-37 levels was performed using [human LL-37 ELISA Kit (Hycult ®)] according to the manufacturer procedure. Final concentrations were based on a standard curve and are shown in ng/ml.

For normalization we divided a urine LL-37 concentration in “ng/ml” by creatinine in “mg/ml” to determine normalized LL-37 in “ng/mg creatinine” i.e. “ng LL-37/mg creatinine”.

The urinary creatinine (Ucr) levels were analyzed as described previously (32), briefly, we measured urinary creatinine colorimetrically by diluting urine samples 1:20 in dilution buffer from the Creatinine Assay Kit (Creatinine-J. REF. 100,111, SPINREACT, S.A./SAU-Ctra. Santa Coloma, 7 E-17176 SANT ESTEVE DE BAS-Girona, Spain). Urinary creatinine calculated in mg/ml based on a standard curve. The normalized levels of LL-37 were expressed as “ng LL-37/mg creatinine” ratios. Creatinine equation formula [LL-37/UCrX100].

### Statistics

SPSS for window (version 16.0) was used for analyses. Students’-test and χ^2^ were used to compare the continuous variables (when normally distributed) and proportions between the cases and controls, respectively. The levels of LL-37 were not normally distributed and were compared between the cases and controls by Mann-Whitney U test. Logistic and linear regression was performed with UTI (logistic) and log of LL-37 level (linear); these were the dependent variables. Spearman correlation (non-parametric) was performed between the plasma and urine levels. *P* < 0.05 was considered statistically significant.

## Results

During the study period, 197 subjects were initially screened, among these 23 (11.7%) were excluded because they had incomplete data, or, not enough samples. The two groups (87 in each arm) which were enrolled were matched in their basic characteristics, as shown in Table 1. There were 38 (43.7%) and 46 (52.9%), *P* = 0. 288 males in the case and control groups, respectively.

**Table 1:** Comparing number (%) of age and gender between controls and cases with urinary tract infection

Gender	Cases	Controls	P. value
Male	46 (52.9)	38 (43.7)	0.225
Female	41 (47.1)	49 (56.3)	
Age groups			
Children	18 (20.7)	15 (17.3)	0.856
Adults	43 (49.4)	45 (51.7)	
Elderly	26 (29.9)	27 (31.0)	

The common organisms isolated from the 87 cases were *Escherichia coli* (38, 43.7%), *Klebsiella pneumoniae* (16, 18.4%), *Enterobacter cloacae* (4, 4.6%), *Pseudomonas aeruginosa* (4, 4.6%), *Proteus mirabilis* (3, 3.4%), *Acinetobacter baumannii* (3, 3.4%), *Acinetobacter lwoffii* (3, 3.4%), *Klebsiella oxytoca* (2, 2.3%), *Morganella morganii* (2, 2.3%), *Pantoea agglomerans* (3, 3.4%), *Pseudomonas luteola* (2, 2.3%), *Enterococcus faecalis* (2, 2.3%), *Enterococcus faecium* (2, 2.3%), *Staphylococcus aureus* (2. 2.3%), *Staphylococcus saprophyticus* (1, 1.1%).

**Fig. 1:**
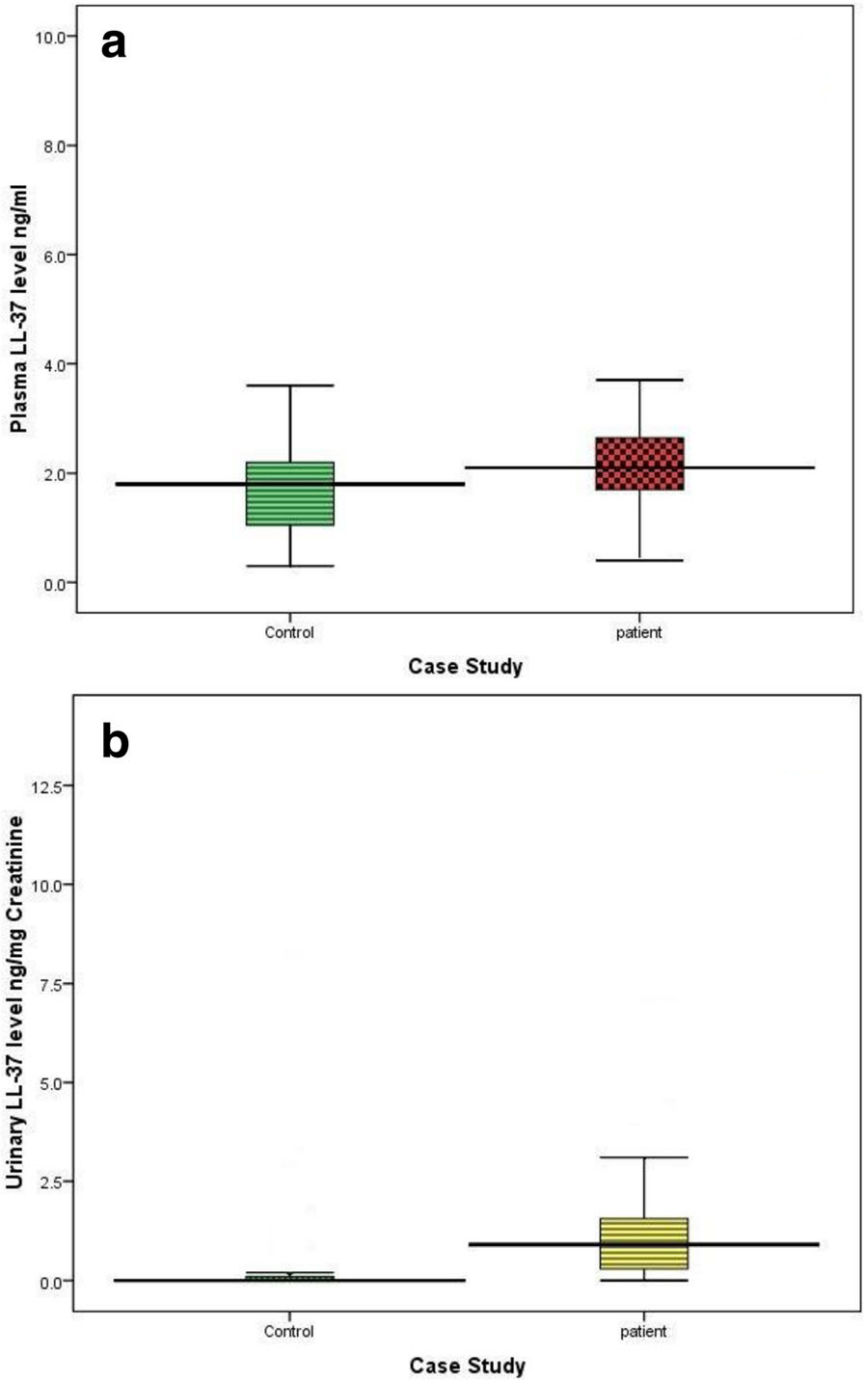
**a** Distribution of plasma LL-37 concentrations in blood samples from culture positive patients with UTI compared to healthy controls participants. The patients have higher levels of plasma LL-37 than controls. **b** Distribution of urinary LL-37 concentrations after normalized to creatinine concentrations in urine samples from culture positive patients with UTI compared to healthy controls participants. The patients have higher levels of urinary LL-37 than controls

Compared with the control group, median (inter-quartile) plasma [2.100 (1.700–2.700) vs. 1.800 (1.000–2.200) ng/ml, *P* = 0.002] and in urine [0.900 (0.300–1.600) vs. 0.000 (0.000–1.000) ng/mg creatinine, *P* < 0.001] LL-37 levels were significantly higher for the cases group (Fig. 1a and b).

There was a significant difference in the plasma and urine levels of LL-37 between males and females when the data for cases and controls were analyzed as a whole or when the data of the controls were analyzed separately. Moreover, in the male cases group, the LL-37 plasma [2.000 (1.675–2.325) vs. 1.800 (0.600–2.200) ng/ml, *P* < 0.001] and urine [0.650 (0.100–1.250) vs. 0.000 (0.000–0.250) ng/mg creatinine, P < 0.001], However, the females had significantly higher median (inter-quartile) plasma LL-37 levels [2.2 (1.800–3.050 vs. 1.900 (1.350–2.200) ng/ml, *P* = 0.001] and urine [0.900 (0.7000–2.050) vs. 0.000 (0.000–0.600) ng/mg creatinine, P < 0.001] LL-37 levels were significantly higher for the female cases group (Fig. 1a and b).

**Fig. 2:**
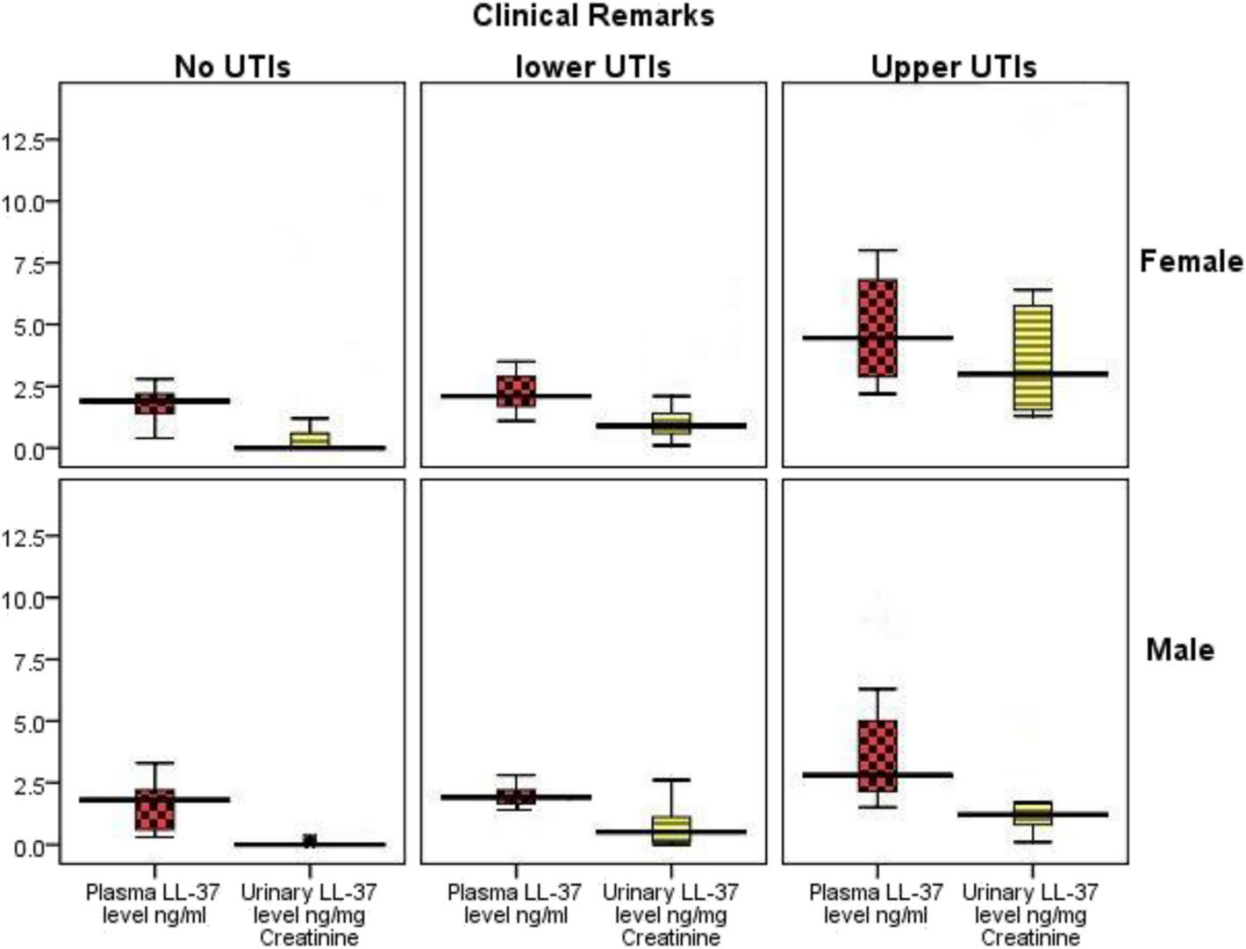
Distribution of plasma and urinary LL-37 levels among patients and control participants according to their clinical remarks (No UTI, Cystitis and Pyelonephritis). The cases with Pyelonephritis has a higher levels of LL-37 in both urine and plasma samples

Compared plasma and urine levels of LL-37 between males and females group, in consideration to the severity of (upper and lower) UTI, median (inter-quartile) plasma [2.000 (1.675–2.325) vs. 2.200 (1.800–3.050) ng/ml, *P* = 0.002] and urine [0.650 (0.100–1.250) vs. 0.900 (0.7000–2.050)] ng/mg creatinine, However, there were no significant difference between males and females in urinary LL-37 levels, a considerable increase of plasma LL-37 was detected among the females (Fig. 2).

There was no significant difference in the median (inter-quartile) plasma [2.1 (1.7–2.9) vs. 2.000 (1.700–2.400) ng/ml, *P* = 0.561] and urine [0.850 (0.275–2.025) vs. 0.900 (0.250–1.350) ng/mg creatinine, *P* = 0.102] LL-37 levels between the *E. coli* isolates and the other isolated organisms.

There was no significant correlation between the plasma and urine level (*r* = 0.221), even when the data of the cases were analyzed separately. According to linear regression analysis, UTI was significantly associated with high plasma and urinary levels of LL-37 Table 2.

**Table 2:** Linear regression analysis of associated factors, log of plasma and urine LL-37 levels

Variable	Log of plasma LL-37 (ng/ml)			Log of urine LL-37 ng/mg creatinine		
	Coefficient	SE	P	Coefficient	SE	P
Age	−0.002	0.001	0.113	0.000	0.002	0.986
Male gender	−0.093	0.040	0.022	−0.109	0.059	0.066
TWBCs	0.018	0.008	0.038	−0.012	0.013	0.358
Neutrophils	−0.001	0.006	0.893	−0.001	0.007	0.913
Lymphocytes	−0.001	0.006	0.812	−0.004	0.007	0.596
Basophiles	0.001	0.008	0.933	−0.007	0.010	0.513
Urinary tract infections	0.153	0.045	0.001	0.162	0.077	0.038
Plasma LL-37 ng/ml	–	–	–	0.238	0.019	< 0.001

## Discussion

The main findings of the current study were the significantly higher levels of both plasma and urinary LL-37 levels among patients with UTI compared with those of controls. Our findings are in agreement with recent reports [8, 33]. Nielsen et al., reported that, the urinary LL-37 levels were significantly higher during infection than post infection, yet the post infection LL-37 levels were significantly lower in patients with UTI than those of controls [32]. In another setting, Ovunç Hacıhamdioglu D. et al., showed no significant differences in the levels of urinary LL-37 between the children with UTI and the control groups [34].

In previous reports, the activity and functions of some other biomarker compounds have been mentioned, as for example, the antibacterial properties of the epithelial cells plays a role in the innate immunity of the urinary tract [35]. Moreover, it has been shown that some factors of innate immunity (Tamm-Horsfall protein) interfere with bacterial adherence [36], sIgA antibodies inhibit bacterial colonization [37], and inhibit bacterial growth [38] or directly kill uropathogens [39, 40]; the bacterial attachment results in exfoliation of host bladder epithelial cells [41].

Similar results were shown by Chromek et al. who studied urinary cathelicidin from both healthy children and children with UTIs and observed that cathelicidin is constitutively expressed in the urinary tract. Milan Chromek and his colleges mentioned that, the direct contact with microbes induces urinary epithelial cells to substantially increase production and secretion of cathelicidin, protecting the urinary tract from microbial adherence. Cathelicidin deficiency predisposes to urinary infections as it has been shown that mice which are CRAMP-deficient (Camp−/−) are more susceptible to UTI than CRAMP-producing (Camp+/+) mice [31].

**Fig. 3:**
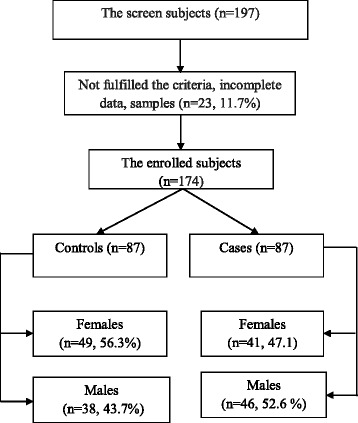
Schematic diagram of the study design

Our findings provide the first evidence in adult and young children of both genders that the level of LL-37 correlates with positive urine culture in cases of urinary infections. Furthermore, LL-37 levels can be used as a marker of infection and could save time in confirming an accurate diagnosis of UTI in the acute phase. The ability to make an early decision about whether there is an infection or not and would be helpful in limiting unnecessary antimicrobial administration for suspected urinary infections, see Fig. 2. In turn, this would decrease health care costs and the problems associated with inappropriate use of antibiotics. Our results demonstrated differences in plasma and urinary levels of LL-37 between subjects with and without UTIs. To the best of our knowledge, this is the first study performed in apparently healthy and non-hospitalized controls that compares their plasma and urine LL-37 levels with patients (both genders) with UTIs. Additionally, this is the first study to compare inter-individual baseline plasma and urine LL-37 levels for patients and for controls. Table 2, Figs. 1a and b and 3.

In the current study there was no significant difference in both plasma and urine LL-37 levels when comparing isolates of *E. coli* and other uropathogens. This indicates that the microbial infection increases the levels of LL-37 in both plasma and urine concurrently.

To determine the exact circulating level of LL-37 in plasma in relation to UTIs further research needs to be conducted. Studies with much larger subject numbers are required. The limitation of the current study; there was no follow-up sampling to detect LL-37 level after clearance of infection.

## Conclusion

Infection of the urinary tract increases the levels of LL-37. The observed increased level was not only in the patient’s urine, but has also been observed in the plasma of patients during the period of infection of the urinary tract. Detection of increased levels of LL-37 could help to differentiate subjects with suspected UTI. Accordingly, LL-37 could act as a good marker for diagnosing UTIs.

## Abbreviations

(>): Greater than
(≥): Greater than or equal
(°C): Degrees Celsius
(Camp−/−): CRAMP-deficient
(Camp+/+): CRAMP-producing
(P=): *P*-value
(*r* =): Correlation
(sIgA): Secretory IgA antibodies
(vs): Versus
AMPs: Antimicrobial peptides
AST: Antibiotic sensitivity test
AST-N: Gram negative antibiotic sensitivity card
CAMP: Cathelicidin gene
CBC: Complete blood count
CFU/ml: Colony-forming unit per milliliter
CLED: Cysteine lactose electrolyte deficient
CRAMP: Cathelin-related antimicrobial peptide
*E. coli*: *Escherichia coli*
*E. faecalis*: *Enterococcus faecalis*
EDTA: Ethylene-diamine-tetra-acetic acid
ELISA: Enzyme-linked immunosorbent assay
GN card: Gram negative identification card
GP card: Gram positive ID card
GP/AST: Gram positive antibiotic sensitivity card
Greater than; hCAP-18: Human cationic antimicrobial peptide-18
ID card: Identification card
*K. pneumoniae*: *Klebsiella pneumoniae*
kD: Kilodalton
LL-37: Cathelicidin
LPS: Lipopolysaccharides
*M. morganii*: *Morganella morganii*
MIC: Minimum inhibitory concentrations
ng/ml: Nanograms per milliliter
*P. aeruginosa*: *Pseudomonas aeruginosa*
*P. mirabilis*: *Proteus mirabilis*
SPSS: Statistical package for the social sciences
Ucr: urinary creatinine
UPEC: Uropathogenic *E. coli*
UTI: Urinary tract infection
V2C: VITEK-2 Compact System
WBC: White blood cell
χ^2^: Chi-Square
